# Facile Preparation of Polysiloxane-Modified Asphalt Binder Exhibiting Enhanced Performance

**DOI:** 10.3390/polym15183795

**Published:** 2023-09-17

**Authors:** Jinhua Qian, Fuying Dong, Xiaohui Chen, Xianying Xu, Dongkang Zhang, Fulong Li, Yuxia Gao, Huadong Sun, Laixue Pang, Xinde Tang, Dengxu Wang

**Affiliations:** 1 School of Traffic and Civil Engineering, Shandong Jiaotong University, Jinan 250357, China; 2 Institute of Intelligent Transportation, Shandong Jiaotong University, Jinan 250357, China; 3 National Engineering Research Center for Colloidal Materials & Key Laboratory of Special Functional Aggregated Materials, Ministry of Education, School of Chemistry and Chemical Engineering, Shandong University, Jinan 250100, China

**Keywords:** polysiloxane, modified asphalt, rheological property, interaction mechanism, polymer

## Abstract

The development of polymer-modified asphalt (asphalt = asphalt binder) is significant because the polymer modifier can improve the performance of asphalt mixture and meet the requirements of the modern asphalt pavement. Herein, we present a novel polysiloxane-modified asphalt with enhanced performance, formed by simply mixing hydroxy-terminated polysiloxane (HO-PDMS) into base asphalt at 140 °C. The interaction mechanism of HO-PDMS in base asphalt was characterized by FT-IR, GPC, and DSC. It reveals that HO-PDMS polymers have been chemically bonded into the asphalt, and, thus, the resultant asphalt exhibits optimal compatibility and storage stability. The results based on fluorescence microscopy and a segregation test prove that HO-PDMS has good compatibility with base asphalt. Moreover, by virtue of the intriguing properties of polysiloxane, the present asphalt possesses improved low- and high-temperature properties, higher thermal stability, and enhanced hydrophobicity compared to conventional asphalt when using an appropriate dosage of HO-PDMS. DSC indicated that the *T_g_* of modified asphalt (−12.8 °C) was obviously lower than that of base asphalt (−7.1 °C). DSR shows that the rutting parameter of modified asphalt was obviously higher than that of base asphalt. BBR shows that modified asphalt exhibited the lowest stiffness modulus and the highest creep rate with an HO-PDMS dosage of 6% and 4%, respectively. These results demonstrate that polysiloxane-modified asphalt can be promisingly utilized in realistic asphalt pavement with specific requirements, particularly high-/low-temperature resistance.

## 1. Introduction

The mileage of high-grade highways built in China has become number one in the world, and over 95% of these highways are built using asphalt pavement. However, the distressing of asphalt pavement, such as the presence of ruts, cracks, and water damage, is becoming more serious with the increasing traffic load, decreasing the service life and hindering the sustainable development of highways. Meanwhile, there are stringent requirements for the performance of asphalt pavement for special-application scenarios, such as high-speed toll gates, airport runways, steel bridge decks, and harsh geographical environments.

To address the above-mentioned problems of asphalt pavement, asphalt can be prepared by doping modifiers through physical blending or chemical grafting [1,2,3,4]. Numerous engineering applications have proven that using a polymer to modify asphalt is the most effective way to solve these problems. The viscoelastic property, rutting resistance, and cracking resistance of asphalt pavement can be enhanced significantly by adding a polymer modifier [5,6,7]. At present, polymer modifiers for asphalt primarily include styrene-butadiene-styrene triblock copolymer (SBS) [8], styrene butadiene rubber [9], waste rubber powder [10], polyethylene [11], vinyl acetate-vinyl copolymer [12], and epoxy resin [13]. In particular, SBS has an obvious advantage in improving the low- and high-temperature properties of asphalt; thus, it is extensively used in asphalt-pavement engineering. However, when facing the severe challenges of the deterioration of the traffic environment in recent years in China, these forms of polymer-modified asphalt still have the following defects: high-temperature softening resistance, low-temperature cracking resistance, and anti-aging. These factors seriously degrade the quality of asphalt pavement [14,15]. Moreover, the preparation of conventional hot-mix asphalt usually requires a high temperature (>160 °C). On one hand, the high temperature seriously reduces the anti-aging performance of asphalt and, thus, shortens its service life. On the other hand, the high temperature leads to high pollution, high energy consumption, and high carbon emissions, thereby seriously damaging the environment and human health, and contradicting the green and low-carbon development concept. Therefore, it is urgent that we develop a novel polymer-modified asphalt to further improve the performance of asphalt mixture, promote the development of asphalt pavement, and meet the design requirements of modern, green, and long-lived asphalt pavement engineering.

Organosilicon material is widely adopted in many fields because of its excellent properties [16,17,18]. It is also used as a modifier in the field of asphalt pavement. As early as 1952, Sanderson [19] found that the adhesion between aggregates and asphalt can be significantly improved by the steam treatment of aggregates with methyl chlorosilane, paving the way for the application of organosilicon in asphalt pavement. In 1973, Schmidt [20] adopted a silane coupling agent to modify asphalt. They found that introducing the silane coupling agent can increase the adhesion between asphalt and aggregates, and the high- and low-temperature resistance of asphalt pavement can be improved. Since then, silane coupling agents have been extensively used to modify various aggregates or fillers (such as basalt fiber [21], fly ash [22], zeolite warm-mix agent [23], and steel slag [24]) to prepare asphalt mixture, and the performance of the modified asphalt pavement has been effectively improved. Silicone resin can also modify asphalt. Compared with base asphalt, silicone-resin-modified asphalt exhibits excellent storage stability and aging resistance. The adhesion between asphalt and aggregates can be strengthened, and even the water damage resistance of asphalt mixture can be enhanced [25,26]. Generally, organosilicon-modified asphalt shows great potential applications in preparing asphalt pavement. Polysiloxane is one of the most important organosilicon materials, and the production capacity of polysiloxane in China accounts for more than 70% of the global market. Moreover, various kinds of polysiloxane with different functional groups such as silanol, vinyl, and amino provide more possibilities for asphalt modification. In particular, the excellent properties of polysiloxane (e.g., heat resistance, cold resistance, aging resistance, and hydrophobicity) fit the requirements of long-lived asphalt pavement. Few works of research have shown that the fatigue resistance and storage stability of asphalt were improved with polysiloxane modification [27,28]. In addition, low-viscosity polysiloxane also exhibits optimal fluidity, which can greatly decrease the viscosity of asphalt and, correspondingly, significantly reduce the processing temperature of asphalt. For example, polysiloxane was used as an ingredient in the warm-mix additive to modify asphalt mixture. The viscosity of asphalt was reduced and the rutting resistance and waterproofing ability were also improved [29]. Thus, choosing polysiloxane as a modifier for preparing asphalt pavement has important research value and application prospects.

In the present study, hydroxy-terminated polysiloxane (HO-PDMS) was selected to modify base asphalt. The silanol groups of HO-PDMS had high activity and can react with themselves or other active functional groups in asphalt. Introducing HO-PDMS into asphalt by chemical cross-linking is expected to improve the compatibility and miscibility of HO-PDMS and asphalt, and a novel kind of polymer-modified asphalt with excellent storage stability could be obtained. The interaction mechanism of HO-PDMS in base asphalt was characterized by Fourier-transform infrared spectroscopy (FT-IR), nuclear magnetic resonance spectroscopy (^1^H-NMR), and gel permeation chromatography (GPC). The effects of HO-PDMS on asphalt properties were comprehensively investigated by physical property tests, differential scanning calorimetry (DSC), a dynamic shear rheological (DSR) test, a bending creep stiffness (BBR) test, thermogravimetric analysis (TGA), and a static contact angle test.

## 2. Materials and Methods

### 2.1. Materials

The base asphalt used in this work was Hongrun 70# (based on penetration), and its basic technical indices are presented in Table 1. HO-PDMS (industrial grade) was a colorless liquid and supplied by Shandong Dayi Chemical Co., Ltd., Laiyang, China. Tetrahydrofuran (THF) was purchased from HEOWNS Biochem Technologies, LLC, Tianjin, China.

### 2.2. Preparation of HO-PDMS-Modified Asphalt

A series of modified asphalt was prepared using HO-PDMS as a modifier. The formulations are shown in Table 2. The experimental procedure of preparation was as follows: Base asphalt was heated to 140 °C at a stirring rate of 700 rpm for 10 min. Then, a stoichiometric amount of HO-PDMS was added into base asphalt for 10 min. Afterwards, the mixture was stirred at 700 rpm for 1 h, and then HO-PDMS-modified asphalt was obtained.

### 2.3. Test Methods

#### 2.3.1. FT-IR

FT-IR (Bruker VERTEX 70, Karlsruhe, Germany) analysis was conducted to determine the functional groups of HO-PDMS-modified asphalt. The test was performed using the potassium bromide pellet method from 400 cm^–1^ to 4000 cm^–1^ at a resolution of 4 cm^−1^.

#### 2.3.2. ^1^H-NMR

^1^H-NMR spectra were obtained with a superconducting nuclear magnetic resonance spectrometer (Bruker AVANCE 400 MHz, Karlsruhe, Germany) using CDCl_3_ as solvent.

#### 2.3.3. GPC

GPC (WATERS 1515-2414, Milford, MA, USA) analysis was conducted to measure the molecular weight of HO-PDMS-modified asphalt. THF served as the mobile phase at a flow rate of 1.0 mL/min.

#### 2.3.4. FM

A fluorescence microscope (ZEISS-Axioscope 5, Oberkochen, Germany) was used to evaluate the compatibility of HO-PDMS with asphalt. Phase structure and morphology were observed at 100× magnification with FM.

#### 2.3.5. Segregation Test

Segregation test was performed according to ASTM D5976. The modified asphalt (about 50 g) was heated and filled into an aluminum tube. It was sealed and placed in an oven at 163 °C for 48 h, and then the sample was placed in a refrigerator to cool for more than 4 h. After dividing the tube into three equal sections, the softening point of the upper and lower samples were measured and denoted as *T_1_* and *T_2_*, respectively. The discrepancy between *T_1_* and *T_2_* was recorded as ∆T.

#### 2.3.6. DSC

DSC (Mettler Toledo DSC 3, Zurich, Swiss) was conducted to measure the glass transition temperature (*T*_g_) and evaluate the low-temperature property of HO-PDMS-modified asphalt. The test was performed at a heating rate of 5 °C/min in nitrogen with 70 mL/min from −50 °C to 300 °C.

#### 2.3.7. Physical Property Tests

Penetration (25 °C), ductility (10 °C), and softening point were tested to evaluate the physical properties of HO-PDMS-modified asphalt according to ASTM D5, ASTM D113, and ASTM D36, respectively.

#### 2.3.8. DSR

The phase angle (δ) and rutting parameter (G*/sin δ) of the modified asphalt were tested by a DSR system (Gemini II ADS, Malvern, UK) according to ASTM D7175. The test was conducted with a strain control mode, the loading frequency was set at 10 rad/s, and the strain was 12%. The initial test temperature was set as 46 °C, and the interval was 6 °C.

#### 2.3.9. BBR

Creep stiffness modulus (*S*) and creep rate (*m*) were measured with a BBR system (TE-BBR-F type, USA) according to ASTM D6648 to evaluate the rheological property of the modified asphalt at low temperatures. The testing temperatures were set at −6 °C, −12 °C, and −18 °C.

#### 2.3.10. TGA

A TGA system (TA TGA55 DISCOVERY, Newcastle, DE, USA) was used to determine the thermal stability of the modified asphalt. The sample was heated in an Al_2_O_3_ crucible from 45 °C to 800 °C at a heating rate of 20 °C/min under nitrogen and air atmospheres, respectively.

#### 2.3.11. Static Contact Angle Measurement

The static water contact angle was measured using the sessile drop method on a contact angle analyzer (SZ-CAMC33, Shanghai, China) equipped with a microscope and illumination system. The volume of water droplet was 1.00 μL, and the liquid speed was 0.5 mm/s.

## 3. Results and Discussion

### 3.1. Interaction Mechanism of HO-PDMS in Base Asphalt

HO-PDMS contains Si-OH groups with high activity at the end of molecules. Asphalt has many active groups, such as -−OH and −COOH. A chemical reaction may occur between the Si-OHs in HO-PDMS or the Si-OH and other active functional groups in asphalt. Consequently, the formed cross-linking network structure inevitably affects the performance of the resulting modified asphalt. Thus, studying the interaction mechanism of HO-PDMS in base asphalt was necessary. The interaction mechanism of HO-PDMS in asphalt was studied by FT-IR, ^1^H-NMR, and GPC. Herein, Asphalt/PDMS-4% was taken as an example.

**Figure 1:**
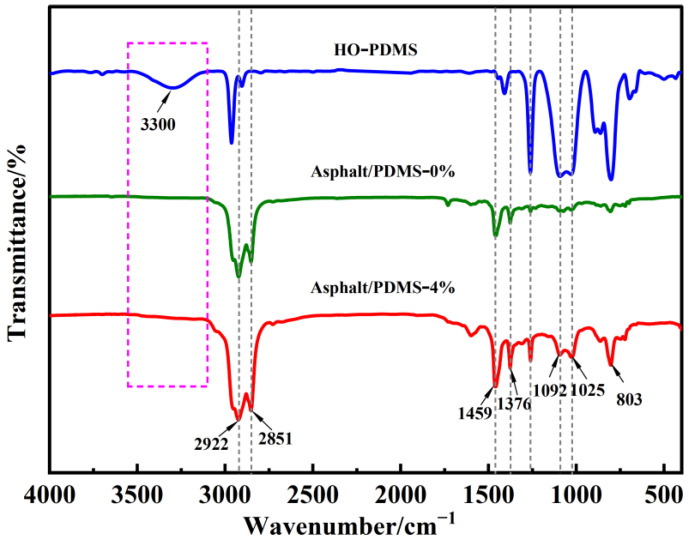
FT-IR spectra of HO-PDMS-modified asphalt.

The FT-IR spectrum of Asphalt/PDMS-4% is shown in Figure 1. The characteristic absorption peak at 1634 cm^−1^ is ascribed to the benzene ring skeleton structure and the telescopic vibration absorption peaks of C=O bonds. The peaks at 1459 and 1376 cm^−1^ are the in-plane and out-of-plane bending vibration absorption peaks of methyl C-H bonds, respectively. The characteristic absorption peaks at 1092 and 1025 cm^−1^ are the telescopic vibration peaks of the Si-O-Si of HO-PDMS, and the telescopic vibration of the Si-C bond is at 803 cm^−1^ [30]. The characteristic absorption peaks of HO-PDMS and base asphalt (Asphalt/PDMS-0%) almost appeared in the FT-IR of Asphalt/PDMS-4%. However, the characteristic absorption peak of Si-OH originating from the HO-PDMS molecules at about 3300 cm^−1^ disappeared in the spectrum of Asphalt/PDMS-4%. This finding indicated that a chemical reaction occurred in preparing the HO-PDMS-modified asphalt. This chemical reaction may have occurred between the Si-OHs in HO-PDMS or the Si-OH and other active functional groups such as −OH or −COOH in asphalt.

**Figure 2:**
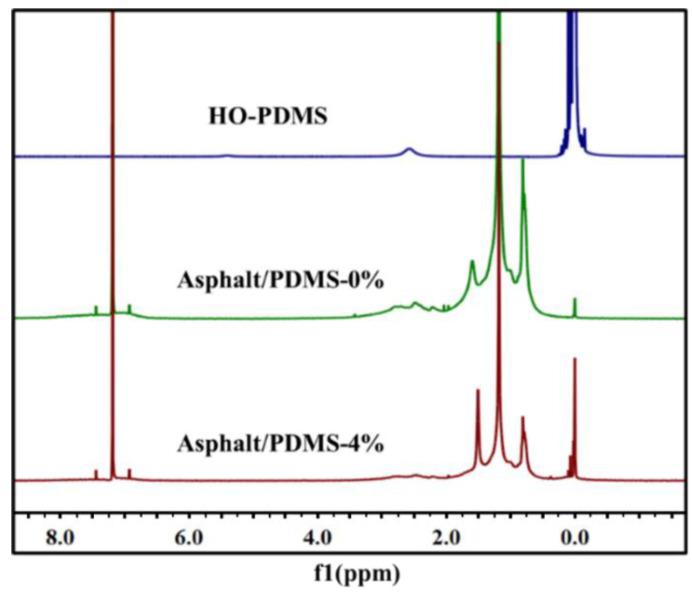
^1^H-NMR spectra of HO-PDMS-modified asphalt.

^1^H-NMR was further used to determine the chemical reaction of HO-PDMS in asphalt. The ^1^H-NMR spectrum of Asphalt/PDMS-4% is shown in Figure 2. In the ^1^H-NMR spectrum of Asphalt/PDMS-4%, the peak at *δ* = 0 ppm was ascribed to the absorption of Si-C*H*_3_, that at *δ* = 2.50 ppm was the absorption of Si-O*H*, and that at *δ* = 7.19 ppm was the absorption of C*D*Cl_3_ solvent. Clearly, the characteristic peaks of HO-PDMS and Asphalt/PDMS-0% appeared in the ^1^H-NMR spectrum of Asphalt/PDMS-4%, whereas no new peaks appeared and the old peaks disappeared in the spectrum of Asphalt/PDMS-4%. This finding implied that the ^1^H-NMR spectrum of Asphalt/PDMS-4% was merely the superposition of the ^1^H-NMR spectrum of HO-PDMS and Asphalt/PDMS-0%. Therefore, determining whether a chemical reaction occurred in HO-PDMS-modified asphalt through the ^1^H-NMR method was difficult.

**Figure 3:**
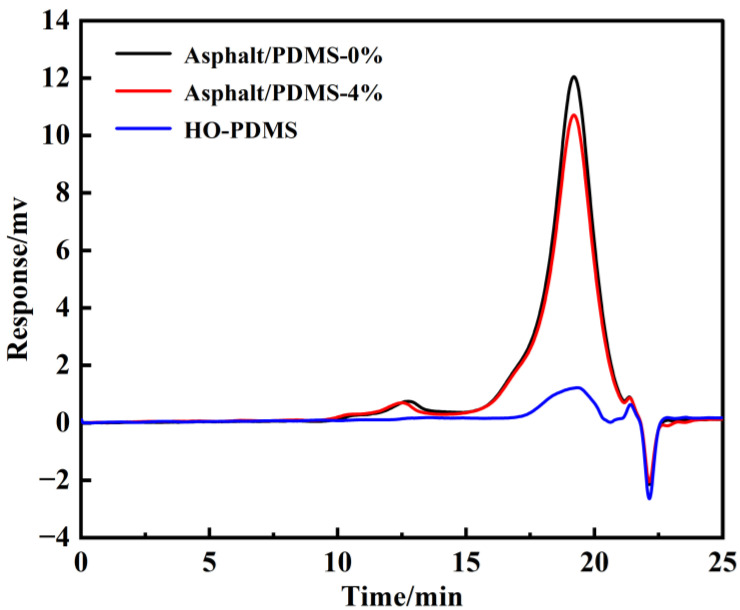
GPC spectra of HO-PDMS-modified asphalt.

The molecular weight of Asphalt/PDMS-4% was characterized by GPC, as shown in Figure 3 and Table 3. The GPC curves of Asphalt/PDMS-0% and Asphalt/PDMS-4% showed two peaks in Figure 3. The left peak was due to the high-molecular-weight substances in asphalt, such as resin and asphaltene. The right peak was due to the small-molecular-weight ones, including the saturated and aromatic components [31]. Compared with Asphalt/PDMS-0%, the number-average molecular weight (*M_n_*) and weight-average molecular weight (*M_w_*) of Asphalt/PDMS-4% increased with the introduction of HO-PDMS. Owing to the low molecular weight of HO-PDMS, the molecular weight of the modified asphalt would decrease if it is only physically mixed, whereas its molecular weight increased with the introduction of HO-PDMS. This result indirectly indicated that a chemical reaction occurred in the process of preparing HO-PDMS-modified asphalt, thereby resulting in the increased molecular weight of asphalt.

**Scheme 1:**
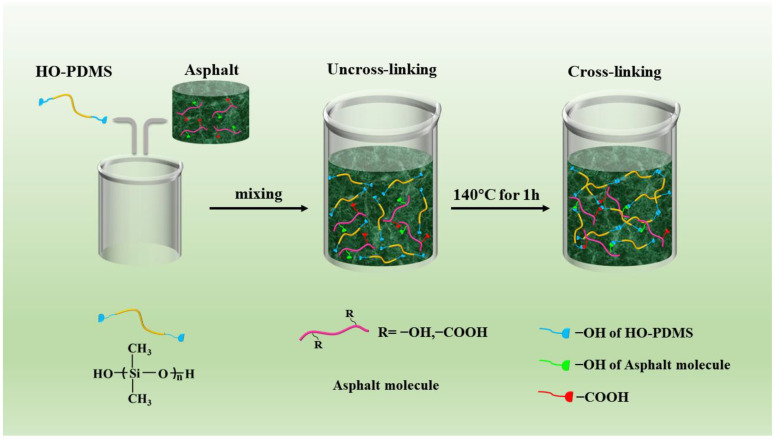
Interaction mechanism of HO-PDMS in base asphalt.

The above test results indicated that the HO-PDMS molecules were chemically bonded into the asphalt during processing and not only physically mixed. The interaction mechanism of HO-PDMS in base asphalt is depicted in Scheme 1.

### 3.2. Compatibility of HO-PDMS with Asphalt

The compatibility and dispersion of the modifier in base asphalt determines the properties of the modified asphalt. Thus, it is most important to studying the compatibility of HO-PDMS with base asphalt and the dispersion of HO-PDMS in asphalt.

**Figure 4:**
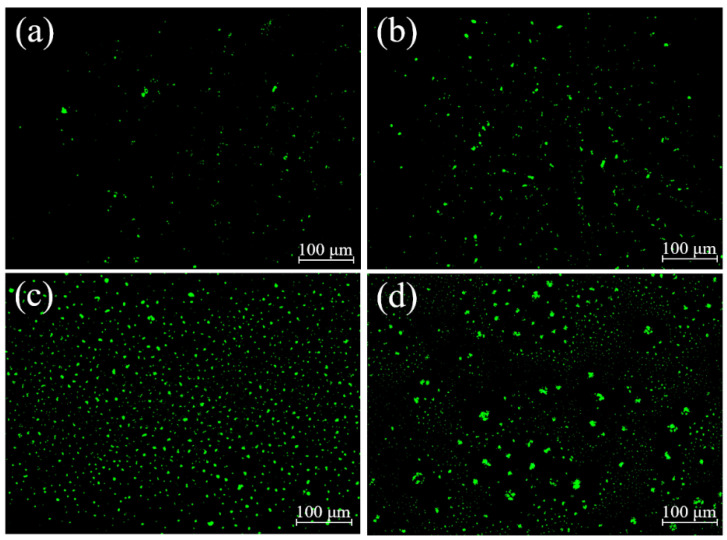
FM of modified asphalt with different HO-PDMS dosages: (**a**) Asphalt/PDMS-2%, (**b**) Asphalt/PDMS-4%, (**c**) Asphalt/PDMS-6%, and (**d**) Asphalt/PDMS-8%.

The effect of HO-PDMS dosage on the compatibility and dispersion of the modified asphalt was investigated by FM. The results are shown in Figure 4. The size and shape of the fluorescent particles reflected the dispersion of the HO-PDMS molecules in asphalt. Figure 4 shows that the fluorescent particles in all measured samples were distributed uniformly, indicating that HO-PDMS with different dosages can be evenly distributed in asphalt. Furthermore, the density of the fluorescent particles in asphalt notably increased with an increased HO-PDMS dosage, and the particle size was basically consistent, as shown in Figure 4a–c. This finding indicated that modified asphalt with good compatibility can be obtained as the HO-PDMS dosage increases to 6%. However, the particle size was larger and the dispersion density of the fluorescent particles decreased when increasing the dosage of HO-PDMS to 8% (Figure 4d). This result was ascribed to the increase in HO-PDMS intermolecular collision with the excessive HO-PDMS dosage [32]. It has been demonstrated that this chemical reaction occurred between the Si-OHs in HO-PDMS or the Si-OH and other active functional groups in asphalt from the result of FT-IR, ^1^H-NMR, and GPC. With the increase of HO-PDMS content, more and more Si-OHs in HO-PDMS reacts, resulting in the serious aggregation of HO-PDMS molecules in asphalt.

**Figure 5:**
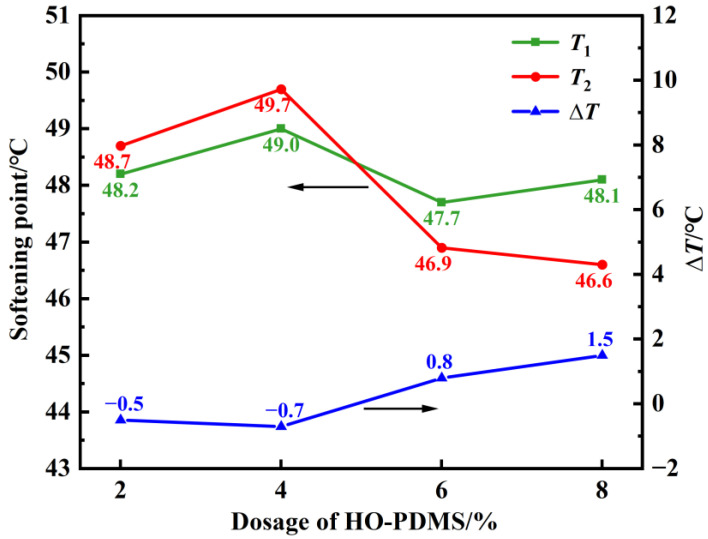
Softening point of modified asphalt conducted after segregation test.

To further investigate the effect of HO-PDMS dosage on compatibility, the softening point of the sample was measured after it was subjected to a segregation test. Figure 5 shows that the discrepancy of the softening point (∆*T*) of the measured modified asphalt were all less than 2.2 °C, indicating that the HO-PDMS-modified asphalt was not prone to aggregate according to ASTM D5976. It denotes that the modified asphalt exhibited satisfactory storage stability at high temperatures. Theoretically, HO-PDMS has low surface tension, which is unfavorable to its compatibility with asphalt. Actually, HO-PDMS exhibited optimal compatibility with asphalt in this work, which we ascribed to the chemical reaction of HO-PDMS in asphalt. Figure 5 also shows that the ∆*T* of the sample Asphalt/PDMS-8% was 1.5 °C, which was much higher than that of other samples. This finding implied that the dispersion of HO-PDMS in asphalt worsened with an increment of the HO-PDMS dosage to 8%, in agreement with the FM result.

### 3.3. Low-Temperature Property

**Figure 6:**
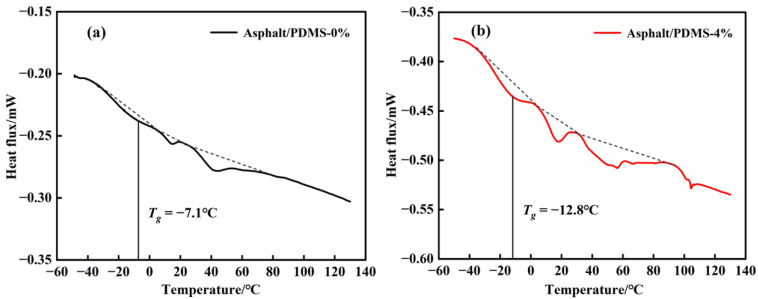
DSC curves of Asphalt/PDMS-0% (**a**) and Asphalt/PDMS-4% (**b**).

DSC analysis was performed to study the low-temperature property of HO-PDMS-modified asphalt. The DSC curves of modified asphalt are shown in Figure 6. The *T_g_* of Asphalt/PDMS-4% (−12.8 °C) was obviously lower than that of Asphalt/PDMS-0% (−7.1 °C). The main chain of polysiloxane comprised Si-O-Si bonds. The bond length of the Si-O-Si bond was longer than that of C-C, C-O-C, and other chemical bonds in carbon-based compounds. Correspondingly, the molecular chain of polysiloxane had excellent flexibility at low temperatures, and the *T_g_* of polysiloxane was lower than −120 °C [33,34]. Therefore, the introduction of HO-PDMS can increase the flexibility of asphalt at low temperature and, consequently, lower the *T_g_* of asphalt, thereby improving the low-temperature resistance of asphalt.

**Figure 7:**
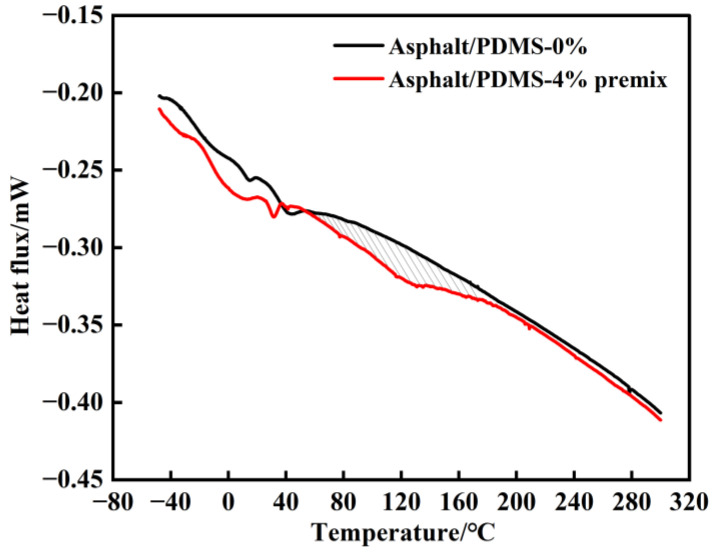
DSC curves of Asphalt/PDMS-0% and Asphalt/PDMS-4% premix.

According to the formulation of the Asphalt/PDMS-4% sample listed in Table 2, a stoichiometric amount of HO-PDMS and base asphalt were dissolved in THF at room temperature, mixed evenly, and dried in a vacuum for 24 h. A premix of HO-PDMS-modified asphalt was finally obtained. The DSC curve of the premix was measured from −50 °C to 300 °C, as shown in Figure 7. Compared with the base asphalt, the premix had an endothermic peak at 60 °C to 180 °C, whereas the reaction of the Si-OH in HO-PDMS with other active groups was within this temperature range [35,36]. Therefore, the DSC measurement result further proved that a chemical reaction occurred in preparing modified asphalt added with HO-PDMS.

### 3.4. Physical Properties

**Figure 8:**
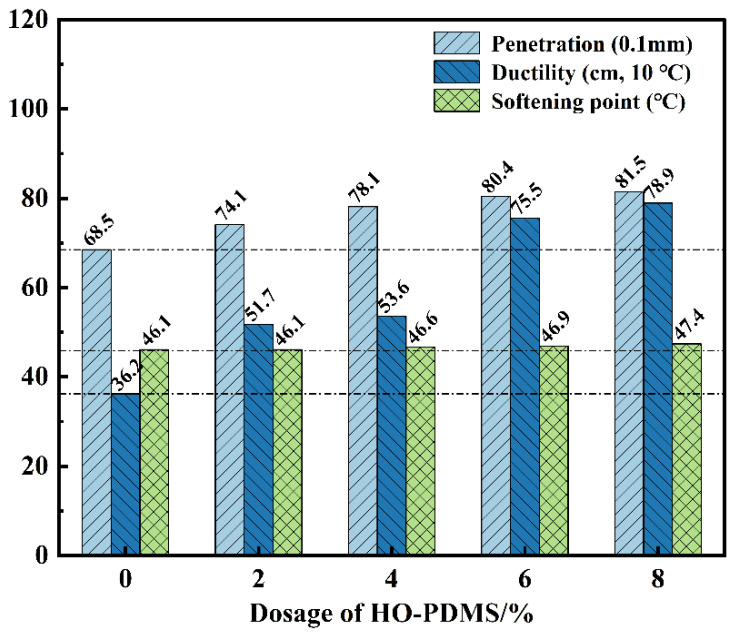
Effects of HO-PDMS dosage on the physical properties of asphalt.

Physical properties including the penetration, ductility, and softening point were measured to evaluate the hardness, low-temperature performance, and high-temperature performance of HO-PDMS-modified asphalt. As shown in Figure 8, the penetration and ductility of the modified asphalt increased with an increment of the HO-PDMS dosage. The increased penetration indicated that the introduction of HO-PDMS can improve the flexibility of the asphalt. The obvious increase in ductility indicated that HO-PDMS can increase the flexibility of asphalt at low temperatures. Thus, the HO-PDMS addition effectively enhanced the flexibility and performance of asphalt at low temperature, consistent with the DSC result due to the excellent flexibility of polysiloxane. Moreover, Figure 8 shows that the softening point of the modified asphalt underwent little change with an increment of the HO-PDMS dosage, indicating that adding HO-PDMS did not adversely affect the high-temperature performance of asphalt.

### 3.5. Rheological Properties

#### 3.5.1. High-Temperature Rheological Property

**Figure 9:**
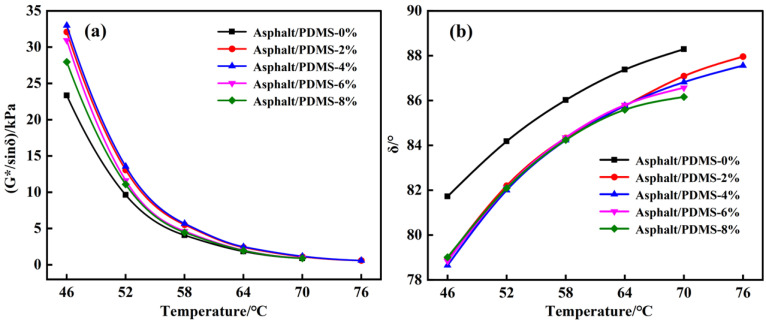
DSR results of HO-PDMS-modified asphalt: (**a**) G*/sinδ, (**b**) δ.

The high-temperature rutting resistance of asphalt pavement can be evaluated with the high-temperature rheological property of asphalt. The high-temperature rheological property of HO-PDMS-modified asphalt was determined by DSR. The results of the rutting parameter (G*/sinδ) and phase angle (δ) are shown in Figure 9. The G*/sinδ of all samples showed a similar trend of a sharp decline first, followed by a gradual decline as the temperature increased, as shown in Figure 9a. This finding indicated that the fluidity of asphalt increased with increased temperature, which was unfavorable to the high-temperature rutting resistance of asphalt [37]. Figure 9a also shows that the G*/sinδ of HO-PDMS-modified asphalt was obviously higher than that of base asphalt (Asphalt/PDMS-0%), especially at low temperatures. This finding indicated that the sensitivity of asphalt to temperature decreased after the modification with HO-PDMS, and the rheological property of the modified asphalt stabilized in response to the change in ambient temperature in practical application. Moreover, Figure 9b shows that the δ of base asphalt and the modified asphalts all increased with increased temperature; i.e., the viscous component of the modified asphalt increased whereas the elastic component decreased with increased temperature. However, the δ of the modified asphalts were all significantly lower than that of base asphalt within the measured temperature range, indicating that adding HO-PDMS increased the viscoelasticity of asphalt at high temperatures. We speculated that the chemical reaction of HO-PDMS in base asphalt helped it form a stable network structure, thereby improving the high-temperature rheological property of asphalt. Therefore, the high-temperature rutting resistance of asphalt increased with the addition of HO-PDMS.

#### 3.5.2. Low-Temperature Rheological Property

**Figure 10:**
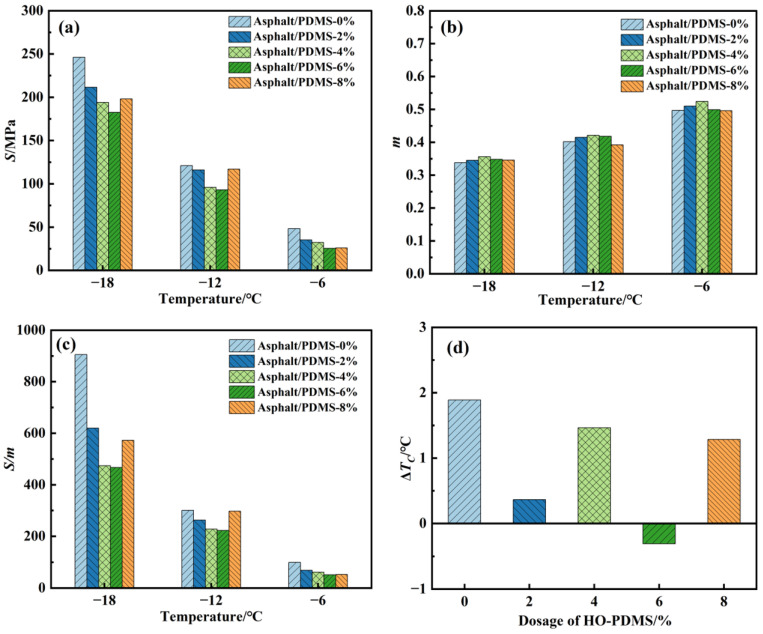
BBR results: (**a**) *S*, (**b**) *m*, (**c**) *S*/*m*, and (**d**) Δ*T_c_*.

The low-temperature rheological property of asphalt is closely related to the low-temperature cracking resistance of asphalt pavement. To study the effect of HO-PDMS on the low-temperature rheological property of asphalt, a BBR test was performed. Figure 10a,b show that the stiffness modulus (*S*) values of all measured samples were less than 300 MPa, and the creep rate (*m*) values all exceeded 0.3 at the three test temperatures of −6 °C, −12 °C, and −18 °C, respectively. This result indicated that the low-temperature rheological property of all samples complied with the requirements of the AASHTO T313 standard. Figure 10a shows that all *S* values measured at the three different temperatures initially decreased, but subsequently increased with an increased HO-PDMS dosage, respectively. The *S* values of all modified asphalts were all lower than that of base asphalt at the same temperature. The modified asphalt exhibited the lowest *S* value when the dosage of HO-PDMS reached 6% (Asphalt/PDMS-6%), and their *S* values decreased by 25.8%, 23.1%, and 47.2% at −18 °C, −12 °C, and −6 °C compared with those of base asphalt, respectively. Figure 10b shows that *m* initially increased and then decreased with an increased HO-PDMS dosage, and Asphalt/PDMS-4% showed the highest *m*. The *S*/*m* value is often used to characterize the low-temperature cracking resistance of asphalt [38]. Figure 10c shows that *S*/*m* initially decreased and then increased with an increased HO-PDMS dosage at the three test temperatures. When the dosage was 4% or 6%, the *S*/*m* value of modified asphalt was lower. However, with further increased content (Asphalt/PDMS-8%), *S*/*m* increased due to the agglomeration of HO-PDMS molecules in asphalt (Figure 4), which adversely affected its low-temperature rheological property. Thus, the results of *S*, *m*, and *S*/*m* indicated that the low-temperature rheological property of asphalt significantly improved with the HO-PDMS addition at an appropriate dosage. Figure 10 also shows that *S* and *S*/*m* decreased and *m* increased with increased temperature. This finding indicated that low temperatures had a negative impact on the low-temperature rheological property of asphalt because the movement of the polymer molecular chain became limited with decreased temperature. Additionally, asphalt underwent partial physical hardening, leading to the deterioration of the low-temperature performance of asphalt. Therefore, taking into account *S*, *m*, and *S*/*m* at −6 °C, −12 °C, and −18 °C, HO-PDMS-modified asphalt with 4% or 6% content had a better low-temperature rheological property, consistent with the results of DSC and ductility tests. ∆*T_c_* was also used to evaluate the durability of asphalt for asphalt pavement [39]. The small absolute value of ∆*T_c_* denotes the asphalt has a good relaxation ability, and then the asphalt pavement would exhibit good crack resistance and durability. Figure 10d shows that the ∆*T_c_* of HO-PDMS-modified asphalt were all lower than that of base asphalt. Finally, the results of *S*, *m*, *S*/*m* and ∆*T_c_* demonstrated that the low-temperature cracking resistance of asphalt pavement can be improved through modification with HO-PDMS.

### 3.6. Thermal Stability

**Figure 11:**
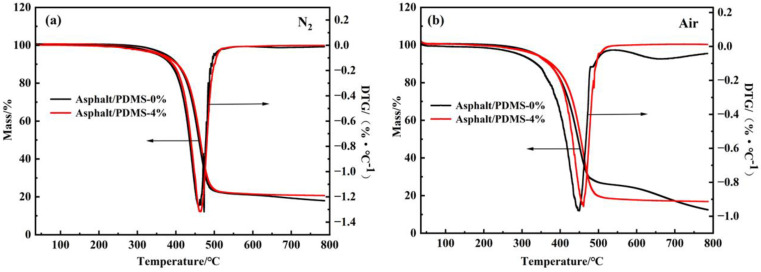
TG and DTG curves: (**a**) in nitrogen atmosphere, and (**b**) in air atmosphere.

TGA was conducted to evaluate the thermal stability of HO-PDMS-modified asphalt under nitrogen and air, respectively. The results are shown in Figure 11. Figure 11a shows that the thermal degradation behavior of Asphalt/PDMS-4% was the same as that of Asphalt/PDMS-0% in a nitrogen atmosphere, of which only one TG loss stage can be found. This finding indicated that adding HO-PDMS did not affect the thermal degradation behavior of base asphalt. The thermal degradation characteristics of Asphalt/PDMS-4% are listed in Table 4. The 50% TG loss temperature (*T*_50_) and maximum degradation rate temperature (*T*_P_) of Asphalt/PDMS-4% were higher than those of Asphalt/PDMS-0%. Furthermore, the residual yield of Asphalt/PDMS-4% (18.1%) was higher than that of Asphalt/PDMS-0% (17.7%) at 800 °C. These results indicated that the thermal stability of Asphalt/PDMS-4% was better than that of base asphalt, which was ascribed to the higher bond energy of the Si-O (443 kJ/mol) in HO-PDMS than C-C (332 kJ/mol) and C-O (326 kJ/mol) [40]. However, Table 4 also shows that the initial degradation temperature (*T*_5_) of Asphalt/PDMS-4% was slightly lower than that of Asphalt/PDMS-0%. The higher mass loss of Asphalt/PDMS-4% within this low-temperature range may be due to the reaction of the Si-OH in asphalt which has been proven by the DSC test.

In an air atmosphere, a second degradation peak appeared in the TG curve of Asphalt/PDMS-0% above 550 °C, as shown in Figure 11b. The oxygen in air promoted the further degradation of Asphalt/PDMS-0% at high temperatures. Conversely, this degradation peak was not found in the TG curve of Asphalt/PDMS-4%, indicating that adding HO-PDMS affected the thermal oxidation degradation behavior of base asphalt. Table 4 shows that the *T*_50_ and *T*_P_ of Asphalt/PDMS-4% were both higher than those of Asphalt/PDMS-0%. The residual yield of Asphalt/PDMS-4% (16.5%) was higher than that of Asphalt/PDMS-0% (12.1%) at 800 °C. This result indicated that the introduction of HO-PDMS effectively inhibited the oxidation degradation of asphalt.

**Figure 12:**
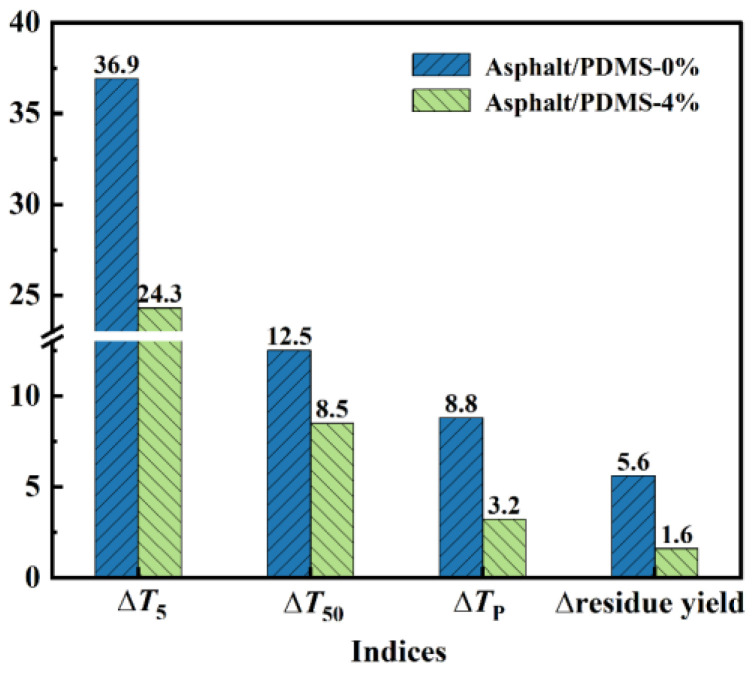
Decomposition indices of Asphalt/PDMS-0% and Asphalt/PDMS-4%.

To further investigate the discrepancy in the effect of HO-PDMS on the thermal stability of asphalt in a nitrogen and air atmosphere, the corresponding decomposition indices including ∆*T*_5_, ∆*T*_50_, ∆*T*_P_, and ∆residue yield were calculated by subtracting the thermal degradation characteristics measured in nitrogen from that measured in air. The decomposition indices are shown in Figure 12. All decomposition indices of Asphalt/PDMS-4% were lower than those of Asphalt/PDMS-0%, indicating that the thermal oxygen degradation property of Asphalt/PDMS-4% was obviously better than that of Asphalt/PDMS-0%. Therefore, the thermal stability of base asphalt significantly improved after the modification with HO-PDMS. In particular, in an air atmosphere, HO-PDMS-modified asphalt showed superior thermal stability.

### 3.7. Surface Property

**Figure 13:**
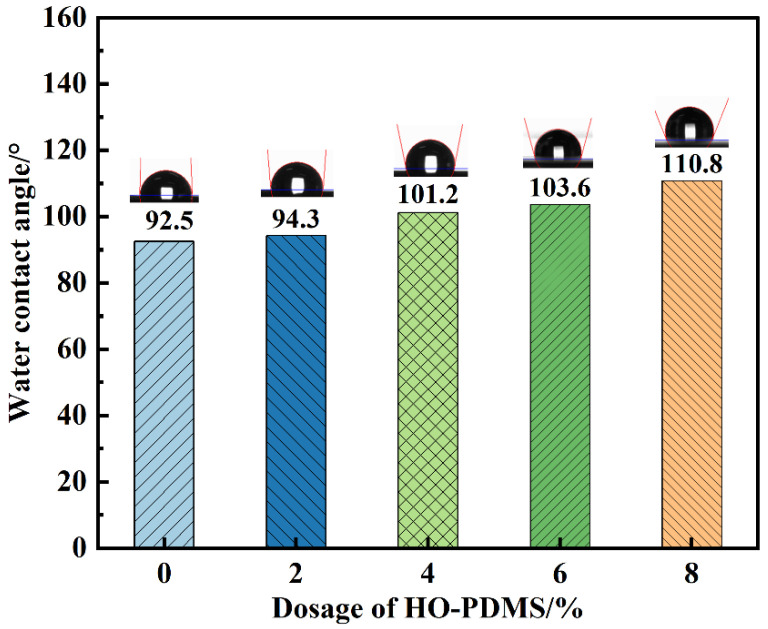
Water contact angle of HO-PDMS-modified asphalt.

The waterproof performance of asphalt pavement was closely related to the surface property of asphalt. The effect of HO-PDMS on the surface property of asphalt was investigated through a water contact angle test. Figure 13 shows that the water contact angle of modified asphalt gradually increased with an increment of the HO-PDMS dosage. When increasing the HO-PDMS content to 8%, the water contact angle reached 110.8°, which was much higher than that of base asphalt (92.5°). This result demonstrated that the hydrophobic property of asphalt was greatly enhanced by introducing HO-PDMS, which was attributed to the unique low surface energy of polysiloxane. Thus, the introduction of HO-PDMS can reduce the surface energy and enhance the hydrophobic property of asphalt, and the waterproof and anti-icing performances of asphalt pavement improved as expected.

## 4. Conclusions

A series of HO-PDMS-modified asphalt was successively prepared by a facile strategy. The HO-PDMS polymers chemically bonded into the asphalt during the preparation of modified asphalt was confirmed by FT-IR, GPC, and DSC analyses. The HO-PDMS-modified asphalt had excellent compatibility and storage stability due to the chemical grafting of HO-PDMS in asphalt. However, the HO-PDMS polymers in asphalt aggregated when the HO-PDMS dosage was excessive. Ductility, DSC, and BBR yielded the consistent result that the low-temperature property of asphalt significantly improved with the addition of HO-PDMS. The high-temperature rheological property was also enhanced with the introduction of HO-PDMS, and the sensitivity of asphalt to temperature decreased after the modification with HO-PDMS at high temperatures. The thermal stability of asphalt in both a nitrogen and air atmosphere increased with the addition of HO-PDMS. The thermal degradation behavior of HO-PDMS-modified asphalt was the same as that of base asphalt in a nitrogen atmosphere. Meanwhile, the thermal oxidation degradation behavior of base asphalt and the modified asphalt in an air atmosphere were inconsistent, and the oxidation degradation of asphalt was effectively inhibited with the addition of HO-PDMS. Furthermore, the hydrophobicity of modified asphalt gradually increased with an increased HO-PDMS dosage due to the low surface energy of HO-PDMS.

Overall, understanding the effects of HO-PDMS as a modifier on the properties of asphalt will provide important information on the application of organosilicon-modified asphalt. And our next work will focus on the evaluation of the performance of HO-PDMS-modified asphalt mixture.

## Figures and Tables

**Table 1:** Technical indices of base asphalt.

Indices	Result	Technical Requirement	Test Standard
Penetration (25 °C, 100 g, 5 s), 0.1 mm	68.5	60–80	ASTM D5
Ductility (10 °C, 5 cm/min), cm	36.2	≥20	ASTM D113
Softening point, °C	46.1	≥46	ASTM D36

**Table 2:** Formulations of HO-PDMS-modified asphalt.

Sample	Base Asphalt/g	HO-PDMS/g
Asphalt/PDMS-0%	100	0
Asphalt/PDMS-2%	100	2
Asphalt/PDMS-4%	100	4
Asphalt/PDMS-6%	100	6
Asphalt/PDMS-8%	100	8

**Table 3:** Characteristic data of GPC.

Sample	*M_n_*/g·mol^−1^		*M_w_*/g·mol^−1^	
Asphalt/PDMS-0%	194,812	1426	288,419	2909
Asphalt/PDMS-4%	251,082	1435	379,093	3038
HO-PDMS	-	1641	-	2626

**Table 4:** Thermal degradation characteristics.

Sample	*T*_5_/°C	*T*_50_/°C	*T*_p_/°C	Residue Yield/%
Asphalt/PDMS-0% (nitrogen)	374.0	460.0	458.8	17.7
Asphalt/PDMS-4% (nitrogen)	356.3	462.3	463.7	18.1
Asphalt/PDMS-0% (air)	337.1	447.5	450.0	12.1
Asphalt/PDMS-4% (air)	332.0	453.9	460.5	16.5

## Data Availability

The data presented in this study are available upon request from the corresponding author.
